# The number and distribution of AMPA receptor channels containing fast kinetic GluA3 and GluA4 subunits at auditory nerve synapses depend on the target cells

**DOI:** 10.1007/s00429-017-1408-0

**Published:** 2017-04-10

**Authors:** María E. Rubio, Ko Matsui, Yugo Fukazawa, Naomi Kamasawa, Harumi Harada, Makoto Itakura, Elek Molnár, Manabu Abe, Kenji Sakimura, Ryuichi Shigemoto

**Affiliations:** 1 Department of Otolaryngology, University of Pittsburgh Medical School, BST3 Building, 3501 Fifth Avenue #10016, Pittsburgh, PA 15261 USA; 2 Department of Neurobiology, University of Pittsburgh, Pittsburgh, PA USA; 3 Center for the Neural Basis of Cognition, University of Pittsburgh, Pittsburgh, PA USA; 4 Division of Interdisciplinary Medical Science, Tohoku University, Sendai, Japan; 5 Department of Brain Structure and Function, Faculty of Medical Sciences, University of Fukui, Fukui, Japan; 6 Max Planck Florida Institute, Jupiter, FL USA; 7 IST Austria, 3400 Klosterneuburg, Austria; 8 Department of Biochemistry, Kitasato University School of Medicine, Sagamihara, Kanagawa Japan; 9 School of Physiology, Pharmacology and Neuroscience, University of Bristol, Biomedical Sciences Building, Bristol, BS8 1TD UK; 10 Niigata University Brain Research Institute, Niigata, Japan

**Keywords:** Electron microscopy, Ventral cochlear nucleus, Synapses, Bushy cells, Fusiform cells, Postsynaptic density, Freeze-fracture replica immunolabeling

## Abstract

The neurotransmitter receptor subtype, number, density, and distribution relative to the location of transmitter release sites are key determinants of signal transmission. AMPA-type ionotropic glutamate receptors (AMPARs) containing GluA3 and GluA4 subunits are prominently expressed in subsets of neurons capable of firing action potentials at high frequencies, such as auditory relay neurons. The auditory nerve (AN) forms glutamatergic synapses on two types of relay neurons, bushy cells (BCs) and fusiform cells (FCs) of the cochlear nucleus. AN-BC and AN-FC synapses have distinct kinetics; thus, we investigated whether the number, density, and localization of GluA3 and GluA4 subunits in these synapses are differentially organized using quantitative freeze-fracture replica immunogold labeling. We identify a positive correlation between the number of AMPARs and the size of AN-BC and AN-FC synapses. Both types of AN synapses have similar numbers of AMPARs; however, the AN-BC have a higher density of AMPARs than AN-FC synapses, because the AN-BC synapses are smaller. A higher number and density of GluA3 subunits are observed at AN-BC synapses, whereas a higher number and density of GluA4 subunits are observed at AN-FC synapses. The intrasynaptic distribution of immunogold labeling revealed that AMPAR subunits, particularly GluA3, are concentrated at the center of the AN-BC synapses. The central distribution of AMPARs is absent in GluA3-knockout mice, and gold particles are evenly distributed along the postsynaptic density. GluA4 gold labeling was homogenously distributed along both synapse types. Thus, GluA3 and GluA4 subunits are distributed at AN synapses in a target-cell-dependent manner.

**Electronic supplementary material:**

The online version of this article (doi:10.1007/s00429-017-1408-0) contains supplementary material, which is available to authorized users.

## Introduction

Diverse information is embedded within the spike trains of each neuron, and the properties of the signals transmitted at individual synapses are at least partially tuned, such that the information encoded in the spike trains can be appropriately transmitted to and interpreted by the postsynaptic target neurons. Neurotransmitters released from presynaptic neurons diffuse to activate their receptors expressed on postsynaptic cell membranes. A range of receptor subtypes has been identified for each neurotransmitter, and these subtypes differ in their molecular organization and pharmacological and biophysical properties, such as their affinity for the transmitter, associated signaling mechanisms, and temporal kinetics of receptor activation and inactivation. Therefore, the type, number, density, and distribution of receptors in a synapse likely shape responses at individual synapses. Here, we identify the differential regulation of the expression of α-amino-3-hydroxy-5-methyl-4-isoxazolepropionic acid (AMPA) receptor subtypes in two different types of postsynaptic neurons that are activated by the same type of presynaptic neurons. We propose that the distinct receptor organization patterns observed in these synapses may underlie the differential retrieval of distinct information from the spike trains, which, in turn, results in the processing of distinct information by these target neurons.

Ionotropic AMPA-type glutamate receptors (AMPARs) mediate fast excitatory transmission along the ascending auditory pathway (Raman et al. 1994). AMPARs are tetrameric complexes composed of homologous or heterologous combinations of GluA1–4 subunits. The electrophysiological properties of the AMPAR channel vary substantially depending on the subunit compositions (Hollmann and Heinemann 1994). For example, GluA1-dominant AMPARs have slow gating characteristics, and these receptors have critical roles in neuronal growth, long-term potentiation, and cognitive functions (Derkach et al. 2007). GluA2-lacking AMPARs are highly permeable to Ca^2+^ and have submillisecond gating kinetics. These receptors are prominently expressed in subsets of neurons that are capable of firing action potentials at high frequencies, such as auditory relay neurons (Geiger et al. 1995; Raman et al. 1994).

The mature auditory nerve (AN) forms synapses with both bushy cells (BCs) of the ventral cochlear nucleus and fusiform cells (FCs) of the dorsal cochlear nucleus. Both synapses contain GluA3 and GluA4 subunits, allowing AMPARs to rapidly respond to released glutamate (Rubio and Wenthold 1997, 1999; Wang et al. 1998; Gardner et al. 1999, 2001; Rubio 2006; Whiting et al. 2009). Compared with AN-FC synapses, AN-BC synapses require extremely rapid synaptic transmission to preserve information contained in the timing of the AN spikes (Gardner et al. 1999; Fujino and Oertel 2003). Thus, the GluA3/GluA4 ratio and the absolute number of these subunits may be specifically tuned at each synapse to meet the demands of the information they must transmit.

The alignment of receptors with presynaptic release sites may influence the probability and timing of receptor activation (Franks et al. 2003; Lisman et al. 2007; Tang et al. 2016). Vesicular release of glutamate in close proximity to high-density AMPAR subdomains (ON cluster release) is likely to elicit larger synaptic responses than glutamate release at low-density AMPAR subdomains (OFF cluster release). Simulations of the dorsal lateral geniculate nucleus (dLGN) have indicated that ON cluster release tends to cause a larger response than OFF cluster release (Tarusawa et al. 2009). Release at the center and periphery of the postsynaptic specialization also appears to produce different response amplitudes. Based on the observations obtained from these simulations, the primary determining factor that governs the amplitude of the synaptic response is the total number of AMPARs expressed, followed by their density. The difference in the response produced by the precise distribution of AMPARs within the postsynaptic specialization appears to be small (Tarusawa et al. 2009). However, the responses produced in the center or periphery may differ, depending on the receptor subunits, which have different response kinetics and dose–response curves. Moreover, the receptor distribution may have a greater influence if release always occurs in close proximity to the AMPAR cluster area or the center or periphery of the synaptic specializations.

Freeze-fracture replica immunogold labeling (FRIL) has been used to determine the localization of receptors within the postsynaptic density (PSD) with extremely high precision. The tangential distribution of synaptic AMPAR subunits has been examined in several synapses in the central nervous system (CNS) using postembedding immunogold methods (Matsubara et al. 1996; Bernard et al. 1997; Jacob and Weinberg 2015). However, the two-dimensional intrasynaptic distribution of AMPAR subunits has only been investigated with FRIL in a few synapses, including dLGN synapses and calyx of Held synapses (Budisantoso et al. 2012, 2013). In the dLGN, two presynaptic terminals from retinogeniculate synapses and corticogeniculate synapses that target onto dLGN relay cells were examined and compared (Tarusawa et al. 2009). Here, we examined AN synapses on BCs and FCs. The same presynaptic spike train likely propagates to both synapses. Thus, the expression of postsynaptic AMPAR subunits and their distribution differ in the two synapses to enable the extraction of specific aspects of information transmitted by the presynaptic AN. The importance of the synaptic architecture may be established by examining the organization of these ultrafast auditory synapses.

## Materials and methods

### Animals used for the morphological analysis

For this study, male CD57B6J wild-type (WT) mice (*n* = 21) and newly developed GluA3-knockout (KO) (*n* = 3) and GluA4-KO (*n* = 3) mice were used at postnatal day 30. The mice were maintained on a 12 h light/dark cycle with water and food available ad libitum. All animal experiments were conducted in accordance with the guidelines of the University of Pittsburgh and Niigata University Animal Care and Use Committees.

### Generation of GluA3 and GluA4 KO mice

Mice deficient in GluA3 or GluA4 were produced by homologous recombination using C57BL/6 embryonic stem (ES) cells (Supplemental Fig. 1). We isolated GluA3 (*Gria3*) and GluA4 (*Gria4*) genes from the C57BI/6 mouse genome using genomic PCR. A GluA3 targeting vector contained exon 11 of the Gria3 gene along with 4.2 kb upstream and 7.0 kb downstream homologous genomic DNA fragments and the diphtheria toxin gene for negative selection. A DNA fragment that carried a loxP sequence and pgk-1 promoter-driven neomycin phosphotransferase gene (Neo cassette) flanked by two Flp recognition target (frt) sites was inserted into the site 107 bp upstream of exon 11. The pgk-1 polyadenylation (poly-A) signal sequence was inserted downstream of the Neo cassette. The other loxP site was introduced into a site 113 bp downstream of exon 11 to eliminate the putative transmembrane domain after Cre-mediated recombination. Homologous recombinant ES clones (*Gria3* ^flox/+^) were identified by Southern blot analysis. The *Eco*RV-digested DNA hybridized with the 5′ probe and yielded a 15.4 kb product for the WT allele and a 14.3 kb product for the targeted allele. The *Nde*I-digested DNA hybridized with the neo probe and yielded a 16.5 kb for the targeted allele; the *Nde*I-digested DNA also hybridized with the 3′ probe and yielded a 14.6 kb for the WT allele and a 16.5 kb product for the targeted allele.

The GluA4 targeting vector contained exon 12 of the *Gria4* gene along with the 8.1 kb upstream and 8.0 kb downstream homologous genomic DNA fragments. The loxP sequence and Neo cassette flanked by two frt sites were inserted into a site 331 bp upstream of exon 12. An internal ribosomal entry site (IRES) sequence and splice donor (SD) sequence from exon 8 of the mouse *Hprt* gene were inserted downstream of the Neo cassette for the poly-A trapping strategy (Shigeoka et al. 2005). The other loxP site was introduced into a site 185 bp downstream of exon 12 to eliminate the putative transmembrane domain after Cre-mediated recombination. Homologous recombinant ES clones (*Gria4* ^flox/+^) were identified by Southern blot analysis. The *Spe*I-digested DNA hybridized with the 5′ probe and yielded a 19.7 kb product for the WT allele and a 9.8 kb product for the targeted allele; the DNA also hybridized with the neo probe to yield a 12.6 kb product for the targeted allele and the 3′ probe to yield a 19.7 kb product for the WT allele and a 12.6 kb product for the targeted allele.

The culture of ES cells and generation of chimeric mice were performed as previously described (Mishina and Sakimura 2007). Briefly, to establish the homologous recombinants, we introduced the linearized targeting vector into the C57BL/6-derived ES lines and subsequently selected recombinant clones with medium that contained 175 μg/mL G418. The targeted clones were microinjected into eight cell-stage embryos of the CD-1 mouse strain. The resulting chimeric embryos were developed to the blastocyst stage by incubating them for more than 24 h and were subsequently transferred to a pseudopregnant CD-1 mouse uterus. Germline chimeras were crossed with C57BL/6 female mice and the heterozygous offspring was crossed with TLCN-Cre mice (Nakamura et al. 2001; Fuse et al. 2004) to establish the GluA3 and GluA4 KO mouse lines.

All animal experiments were conducted in accordance with the guidelines established by the animal welfare committees and the ethics committees of Niigata University.

### FRIL

Mice were anesthetized with ketamine and xylazine and transcardially perfused with 25 mM phosphate-buffered saline (PBS) for 1 min, followed by perfusion with 2% paraformaldehyde (PFA) and a 15% saturated picric acid solution in 0.1 M phosphate buffer (PB) for 12 min. Brains were immediately removed and placed in cold PBS. Coronal slices (130 μm thick) were cut using a vibrating microslicer (DTK-1000; Dosaka EM) in 25 mM PBS. The rostral anteroventral and dorsal cochlear nuclei (AVCN and DCN, respectively) were trimmed from the slice. The trimmed slices were immersed in 30% glycerol/25 mM PBS, incubated overnight at 4 °C and rapidly frozen using a high pressure freezing machine (HPM010; BAL-TEC, Balzers; currently manufactured by RMC Boeckeler Instruments, Tucson, AZ). The frozen samples were then fractured into two parts at −140 °C and replicated by the deposition of carbon (5 nm thick), platinum (uni-direction from 60°, 2 nm), and carbon (20 nm) in a freeze-fracture replica machine (BAF 060; BAL-TEC or JEOL JFDII, or JFDV). After thawing, the tissue debris attached to the replicas was dissolved in a solution containing 15 mM Tris–HCl (pH 8.3), 20% sucrose, and 2.5% SDS with gentle rocking for 18 h at 80 °C. The replicas were subsequently washed with 50 mM Tris-buffered saline (TBS) (pH 7.4) containing 0.05% bovine serum albumin (BSA) and blocked with 5% BSA in washing buffer for 1 h at room temperature (~20 °C). The replicas were incubated with rabbit primary antibodies against GluA1–4 (pan-AMPAR; Nusser et al. 1998), GluA3 or GluA4 (please refer to the “Antibody characterization” section) for 48 h at 15 °C, followed by an overnight incubation with an anti-rabbit [British Biocell International (BBI)] secondary antibody conjugated with 5 nm gold particles at 15 °C. The reliability of the AMPAR localization by FRIL under our fixation conditions has been discussed previously (Tarusawa et al. 2009).

### Antibody characterization

Please refer to Table 1 for a list of all primary antibodies used in the present study. Rabbit polyclonal antibodies against GluA1–4 (pan-AMPAR), GluA3 and GluA4 were used.

**Table 1:** Antibodies used in this study

Antigen	Description of Immunogen	Source, Host species, Cat.#, Clon or Lot	Concentration
GluA1–4 (pan-AMPAR)	Recombinant proteins corresponding to aa 724–781 of rat GluA flop	Raised in Dr. Elek Molnár’s laboratory (Nusser et al. 1998; Pickard et al. 2000). Rabbit, affinity purified polyclonal antibody	3 μg/ml
GluA3	Synthetic peptide of N terminus portion of mouse GluA3 corresponding to aa 394–408, (C)NEYERFVPFSDQQIS	Raised in Dr. Makoto Itakura’s laboratory. Rabbit, affinity purified polyclonal antibody	6.4 μg/ml (FRIL) 0.5–1 mg/l (western blot)
GluA4	Synthetic peptide of N terminus portion of mouse GluA4 corresponding to aa 244–257, (C)FKDISLERFIHGGA	Raised in Dr. Makoto Itakura’s laboratory. Rabbit, affinity purified polyclonal antibody	4.8 μg/ml (FRIL) 0.5–1 mg/l (western blot)

The rabbit anti-AMPAR antibody (anti-GluA1–4 or anti-pan-AMPAR) was raised against a glutathione *S*-transferase (GST) fusion protein that contained the 58 extracellular amino-acid residues (724–781, Table 1) that preceded the last membrane-spanning segment of GluR1*flop* (GST-GluA1flop_(724−781)_). The preparation, purification, and full characterization of this antibody are described in the previous publications (Nusser et al. 1998; Pickard et al. 2000). The antisera were pre-adsorbed with the un-fused GST protein and subsequently affinity purified with the GST-GluA1*flop*_(724−781)_ fusion protein (Pickard et al. 2000). The affinity-purified rabbit polyclonal antibody detected the GST-GluA1*flop*_(724−781)_ fusion protein on immunoblots, and no cross-reactivity to GST was identified (Pickard et al. 2000). In immunoblots of rat brain membranes, this antibody specifically recognized a single band with an approximate size of 110 kDa, which corresponded to the molecular weight of glycosylated AMPAR subunit proteins (Pickard et al. 2000). COS-7 cells expressing individual subunits were used to show that the antibody raised against the conserved extracellular amino-acid residues 724–781 of GluA1*flop* recognized all AMPAR subunits (GluA1–4 *flip* and *flop*), but did not exhibit cross-reactivity with the closely related kainate receptor subunits (Pickard et al. 2000). All immunoreactivity was blocked when the antibody was pre-adsorbed with 100 μg/ml GST-GluA1*flop*_(724−781)_, and no specific staining was detected when the antibody was replaced with the pre-immune serum (Pickard et al. 2000). The selectivity of the pan-AMPAR antibody was further investigated using a guinea pig polyclonal pan-AMPAR antibody raised against GST-GluA1*flop*_(724−781)_ (Pickard et al. 2001). Both the rabbit and guinea pig pan-AMPAR antibodies produced the same staining patterns in rat brain samples (Pickard et al. 2001) and cultured hippocampal neurons (Pickard et al. 2001; Noel et al. 1999). Furthermore, both antibodies immunoprecipitated the same 110 kDa proteins from solubilized rat brain membrane fractions, which were identified on immunoblots as AMPAR subunits using a panel of antibodies selective for the GluA1–4, GluA1, GluA2, and GluA3 proteins (Moult et al. 2006; Gladding et al. 2009). The FRIL patterns obtained with the rabbit anti-GluA1–4 antibody were entirely consistent with our previous reports (Tanaka et al. 2005; Masugi-Tokita et al. 2007; Antal et al. 2008; Tarusawa et al. 2009; Wang et al. 2014; Rubio et al. 2014). Using FRIL, parallel fiber-Purkinje cell synapses of GluA2/3 null mice were not labeled (Masugi-Tokita et al. 2007). Selective immunolabeling have repeatedly been observed in the postsynaptic membrane specialization of various synaptic connections in rat spinal cord (Antal et al. 2008), rat lateral geniculate nucleus (Tarusawa et al. 2009), mouse amygdala (Dong et al. 2010), and rat cochlear nucleus (Rubio et al. 2014). Based on these compelling observations, this antibody specifically labels all four subunits of AMPARs.

The rabbit polyclonal antibodies against GluA3 and GluA4 were raised using keyhole limpet hemocyanin-conjugated synthetic peptides. The following peptides were used: (C)NEYERFVPFSDQQIS is located at the N-terminal extracellular region of rat GluA3 and corresponds to aa 394–408, and (C)FKDISLERFIHGGA is located at the N-terminal extracellular region of rat GluA4 and corresponds to aa 244–257 (Table 1). The antibodies were affinity purified using the synthetic peptides, which were directly coupled to epoxy-activated Sepharose 6B. Synaptosome-enriched (P2) and cytosolic (S3) fractions from the C57B6 mouse brain (without the cerebellum) and the cerebellum were prepared for immunoblotting using a previously described method. Briefly, the brain tissues were homogenized in a buffer containing 5 mM HEPES-NaOH, pH 7.5, and 0.32 M sucrose. The homogenized samples were centrifuged at 800×*g* for 10 min at 4 °C. The supernatant was further centrifuged at 10,000×*g* for 30 min to obtain the pellet, which represented the synaptosome-enriched fraction (P2). The supernatant was centrifuged at 540,000×*g* for 30 min to obtain the supernatant, which represented the cytosolic fraction (S3). Five micrograms of protein from each fraction were separated by SDS–PAGE using a 5–20% gradient gel and analyzed by immunoblotting using the anti-GluA3 antibody or anti-GluA4 antibody (Supplemental Fig. 1). The specificity of the GluA3 and GluA4 antibodies was confirmed by the absence of labeling in replicas obtained from the ventral and dorsal cochlear nuclei of the GluA3 KO and GluA4 KO mice, respectively (Fig. 5).

### Quantification of immunogold particles

Images of excitatory postsynaptic specializations, which were indicated by the presence of intramembrane particle clusters (IMP clusters) on the exoplasmic face (E-face) (Sandri et al. 1972; Harris and Landis 1986) and often accompanied by presynaptic clusters on the protoplasmic face (P-face), were captured at a magnification of 93,000× or 97,000× using a digital camera [MegaView III; Soft Imaging System (SIS) or Orius 830W, Gatan]. IMP clusters were defined as densely packed IMPs at a distance of <15 nm from each other (Tarusawa et al. 2009). The IMP clusters were manually demarcated by connecting the outermost IMP particles, and the areas of individual IMP clusters were measured using the ImageJ software (NIH; RRID: nif-0000-30467). Immunoparticles within demarcated IMP clusters and those located outside and within 30 nm from the edge of the IMP clusters were regarded as synaptic labeling, considering the potential distance between the immunogold particles and antigens (Matsubara et al. 1996). The total number and density of immunogold particles for GluA1–4, GluA3 or GluA4 in each IMP cluster were compared with data obtained from complete synapses. The density of the immunoparticles for GluA1–4, GluA3, or GluA4 in each IMP cluster was calculated by dividing the number of the immunoparticles by the area of the IMP cluster.

### Intrasynaptic distribution of gold particles within the IMP cluster

The distributions of the GluA1–4, GluA3, and GluA4 immunoparticles within the demarcated IMP cluster were initially evaluated by creating a distance map from the border of the demarcation using the FIJI software [distributed under the General Public License (GPL)], as previously described (Budisantoso et al. 2012, 2013). Using this distance map, the IMP cluster area was divided into five divisions by placing contour lines at equal intervals (Figs. 7, 8, 9). An additional division outside of the demarcation (outer rim) with a 30 nm width was also created based on the potential spatial deviation of the immunoparticles from the antigen. The location of each immunoparticle was extracted from this distance map, and the density of immunoparticles in each division was tabulated (Figs. 7, 8, 9).

### Identification of auditory nerve (AN) synapses on the replica

The AN synapses on the replicas of the AVCN and DCN were identified using a previously described method (Rubio et al. 2014). Errors in the identification of AN-BC and AN-FC synapses would imply that the true underlying distributions are even more different than the observed distributions.

#### Identification of AN-BC synapses on replicas of the AVCN

**Fig. 1:**
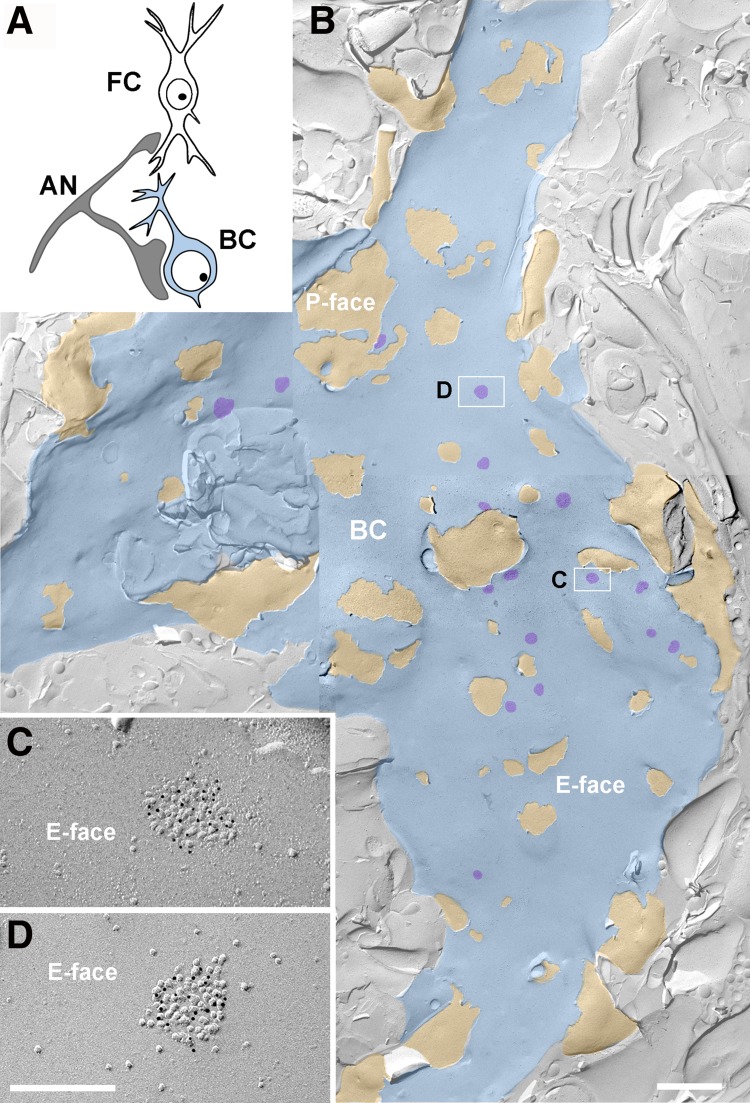
Auditory nerve synapses on bushy cells in the FRIL replica. **a** Schematic of the auditory nerve synapses analyzed with FRIL. *AN* auditory nerve, *BC* bushy cells (*blue color*); *FC* fusiform cells. FRIL electron micrographs at low magnification showing the IMP-cluster distribution of auditory nerve synapses (AN) on bushy cell (BC) soma (**b**). E-face of the BC membrane is pseudocolored in *blue* to aid visualization; P-face of the AN membrane is pseudocolored in *light orange* to aid visualization; IMP-clusters, structural landmark of postsynaptic membrane specialization in replica images, are false colored in *purple* to aid visualization. *Scale bar* 1 μm. **c, d** Images of IMP-clusters (ie; PSDs) of AN synapses on the cell body of a BC immunolabeled with the pan-AMPAR antibody, which reacts with conserved extracellular regions of GluA1–4 (5 nm *gold*), indicating concentration of immunogold particles in IMP-clusters on the E-face. *Scale bar* 200 nm

Only the most rostral sections of the anteroventral cochlear nucleus (AVCN) were used, because this area is enriched with BCs. The auditory nerve forms the main glutamatergic synapse on the cell bodies and dendrites of BCs (Gómez-Nieto and Rubio 2009, 2011; Sento and Ryugo 1989; Ryugo and Sento 1991). Membranes of BC dendrites were rarely observed in the AVCN replicas. In this study, we analyzed the IMP clusters of the AN synapses on the E-face membranes of BC somata (Fig. 1b). The IMP clusters on the E-face membrane of the BC somata were identified as previously described for a rat AVCN replica (Gulley et al. 1977; Rubio et al. 2014).

#### Identification of AN-FC synapses on replicas of the DCN

The DCN is a layered nucleus that is divided into a molecular or superficial layer (ML or layer I), a fusiform cell layer (FCL or layer II), and a deep layer (DL or layers III-IV). The procedure used to identify FCs and their basal dendrites was similar to that used previously (Rubio and Wenthold 1997; Rubio and Juiz 2004). The cell bodies of the FCs are located in the FCL of the DCN and extend their apical and basal dendritic arbors towards the ML and DL, respectively.

### AN-FC synapses

**Fig. 2:**
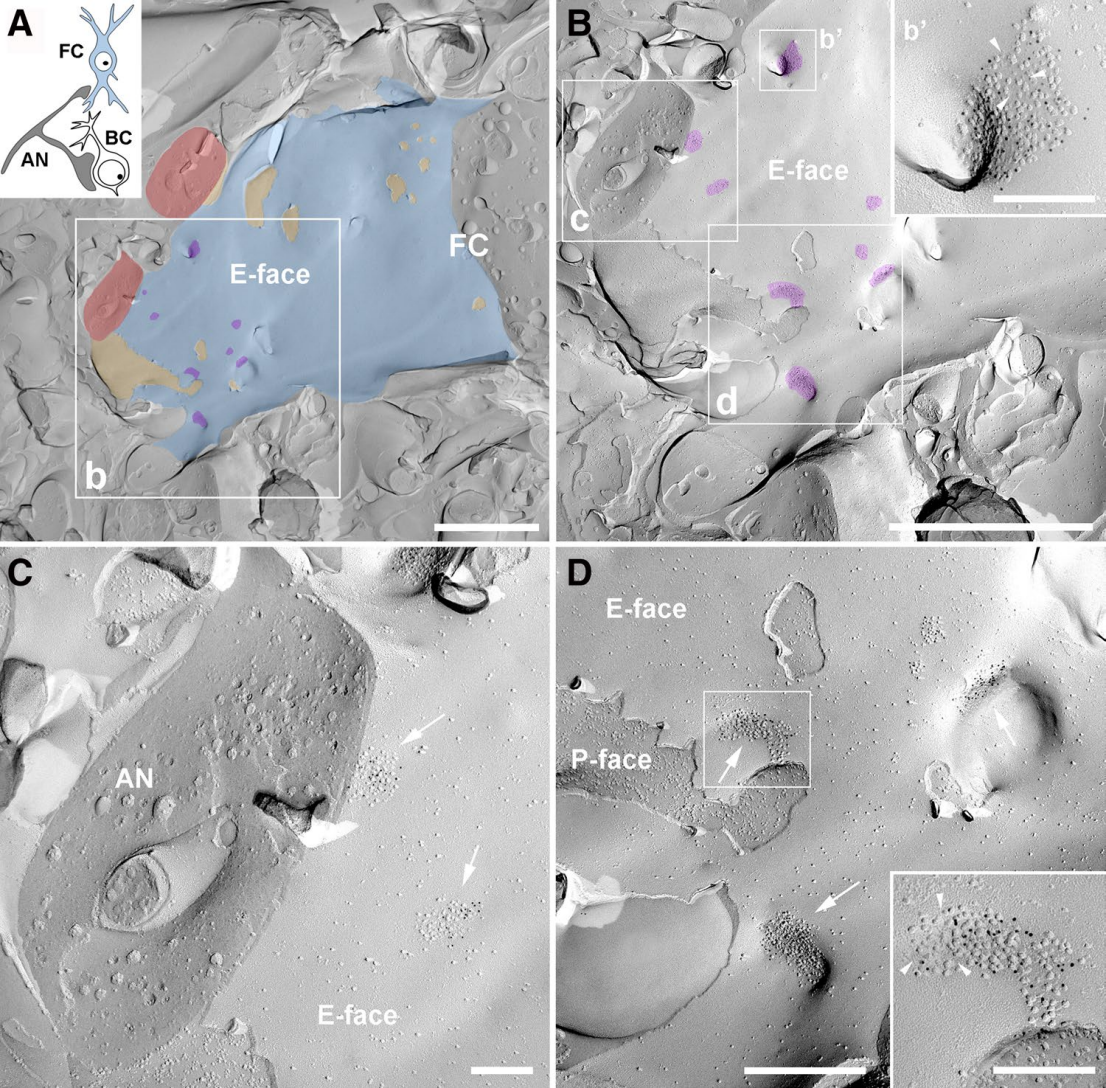
Auditory nerve synapses on fusiform cells in the FRIL replica. **A, B** Schematic of the auditory nerve synapses analyzed with FRIL. *AN* auditory nerve, *BC* bushy cells, *FC* fusiform cells (*blue color*) (*upper left inset*). Low magnification images to show the IMP-cluster distribution of the auditory nerve (AN) synapses on a basal dendrite of a fusiform cell (FC). The E-face of FC membrane at the basal dendrite is pseudocolored in *blue*, the P-face of the AN membrane is pseudocolored in *orange* and cross-fracture auditory nerve profiles (AN) are pseudocolored in *red* and the IMP-clusters are pseudocolored in *purple* to aid visualization. *White boxes* (*b*–*d*) are magnified in **B, C**, and **D**, respectively. *White box* (*b*′) shows higher magnification of an IMP-cluster labeled with gold particles for pan-AMPAR. *Arrowheads* point to a IMP-cluster sub-region that lacked gold labeling. *Scale bars* **A** 2 μm; **B** 2 μm; 200 nm (*b*′ *inset*). **C** Two E-face IMP-clusters (*arrows*) labeled with gold particles for pan-AMPAR and a cross-fracture of a putative auditory nerve (AN). *Scale bar* 200 nm. **D** E-face IMP-clusters (*arrows*) labeled with gold particles for pan-AMPAR. *Inset* shows a higher magnification of the IMP-cluster within the *white box. Arrowheads* point to an IMP-cluster sub-region that lacked gold labeling. *Scale bar* 500, 200 nm (*inset*)

The AN fibers are the primary glutamatergic input within the FCL and DL that contact the FCs (Kane 1974; Smith and Rhode 1985; Ryugo and May 1993; Rubio and Wenthold 1997; Rubio and Juiz 2004). AN inputs form multiple synaptic contacts on the basal pole of the cell body and basal dendrites of FCs (Smith and Rhode 1985; Zhang and Oertel 1994; Rubio and Wenthold 1997). The IMP clusters located on the basal pole of the cell body of identified FCs and the proximal basal dendrites that were identified as extending from the cell body were analyzed (Fig. 2). The IMP clusters on the E-face membrane of the FC basal dendrite were identified as previously described for a rat DCN replica (Rubio et al. 2014).

### Measurement of the width of the postsynaptic membrane specialization from ultrathin sections

WT mice were anesthetized with a ketamine/xylazine mixture and transcardially perfused with 2% paraformaldehyde and 2.5% glutaraldehyde in 0.15 M cacodylate buffer (pH 7.4) with 2 mM calcium chloride at room temperature (~20 °C). The brains were then removed and post-fixed in the same fixative solution for 2 h at 4 °C. Coronal brainstem slices (80 μm thick) were vibratome-sectioned in an ice-cold solution (0.15 M cacodylate buffer and 2 mM calcium chloride). The sections were processed for electron microscopy using a previously described procedure (Rubio et al. 2014).

AN-BC and AN-FC synapses were identified based on their morphological features, as previously described (Rubio and Wenthold 1997; Rubio and Juiz 2004; Gómez-Nieto and Rubio 2009; Rubio et al. 2014). Serial images of the identified synapses were captured from the beginning to the end of each synapse at a magnification of 30,000× using a digital camera. The edge of the PSD was defined as a thickening of the postsynaptic membrane or the presence of a visible synaptic cleft, in addition to the rigid alignment of the presynaptic and postsynaptic membranes. The width and length of the PSD in each section were measured using ImageJ software (http://imagej.nih.gov/ij/). The maximum PSD width in each synapse was used for the analysis.

### Data analysis

All measurements are reported as the means ± standard errors of the means (SEM) unless indicated otherwise. Statistical analyses were conducted using Prism 6 software (GraphPad Software, Inc.), and differences were considered statistically significant at *p* < 0.05. The normality of the data was assessed using Shapiro–Wilk’s *W* test. The statistical evaluation of the immunogold densities was performed using the Mann–Whitney *U* test or Kruskal–Wallis test when appropriate. The statistical evaluation of the maximum PSD and IMP cluster lengths was performed using the Mann–Whitney *U* test. Correlations were assessed using Pearson’s correlation test or Spearman’s rank-order test. One-way ANOVA was used to assess the differences in the intrasynaptic distribution of immunogold particles in each synapse type, and a simple two-way ANOVA test was employed to compare the intrasynaptic distribution between the WT and GluA3 KO mice.

## Results

### Identification of AN synapses on bushy and fusiform cells

**Fig. 3:**
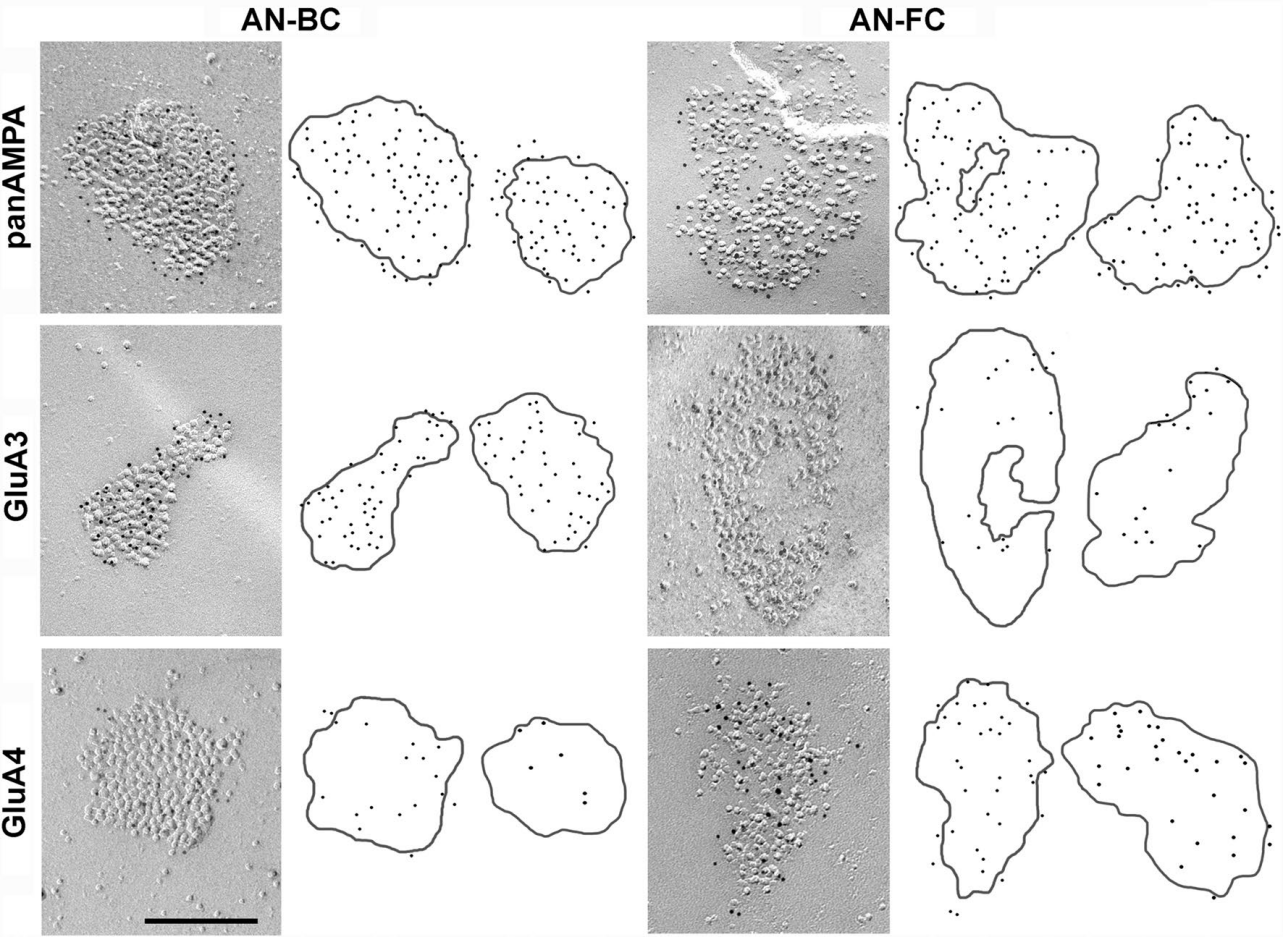
Differential distribution of AMPAR subunits in IMP clusters of AN synapses. FRIL images of IMP-clusters for auditory nerve-bushy cell (AN-BC) and auditory nerve-fusiform cell (AN-FC) synapses that were gold labeled (5 nm gold particles) for pan-AMPAR (GluA1–4), GluA3, or GluA4. The *cartoons on the right* show the distribution of the gold particles for two IMP clusters as representatives. The original size (5 nm) of the gold particles has been enlarged to aid visualization. *Scale bar* 200 nm

The postsynaptic membrane specialization of glutamatergic synapses in a FRIL image is indicated by a cluster of IMPs on the E-face of the plasma membrane (Sandri et al. 1972; Gulley et al. 1977; Harris and Landis 1986) and is often accompanied by the P-face of its presynaptic plasma membrane (Tarusawa et al. 2009; Rubio et al. 2014). The replicas were immunolabeled with specific antibodies against a conserved extracellular region for all AMPAR subunits (GluA1–4) or a peptide sequence unique to GluA3 or GluA4 (Table 1). In the rostral AVCN replicas, the IMP clusters were observed in the putative BC soma and were always immunopositive for the pan-AMPAR, GluA3, and GluA4 antibodies, confirming that these IMP clusters represent the postsynaptic specialization of glutamatergic synapses (Figs. 1, 2, 3). As reported for rats (Rubio et al. 2014), AN-BC and AN-FC synapses were often observed as multiple IMP clusters on large postsynaptic E-face membranes (Figs. 1, 2).

We investigated the synaptic morphology of glutamatergic postsynaptic membrane specializations using FRIL replicas of the rostral AVCN and DCN. We measured the areas of the IMP clusters that were entirely replicated on the fracture plane to compare the sizes of the synapses formed by the ANs on BCs and FCs. The putative PSDs of AN synapses exhibited qualitatively different morphologies in the arrangement of IMPs (Figs. 1, 2, 3). Quantitatively, the IMP cluster area of the AN-BC synapses was much smaller than that of the AN-FC synapses (Table 2, *p* < 0.0001, Mann–Whitney *U* test). Furthermore, we identified round (0.9 circularity; 0.7 roundness) IMP clusters with densely packed IMPs in the AN-BC synapses from the rostral AVCN. Within the DCN, more elliptical (0.6 circularity; 0.6 roundness) IMP clusters with a substantially sparser IMP distribution were frequently identified in the AN-FC synapses (Fig. 3).

**Table 2:** IMP-cluster areas analyzed for AN-BC in the AVCN and AN-FC in DCN

	AVCN	DCN
	AN-BC	AN-FC
Mean	0.031****	0.051
SEM	0.001	0.003
Median	0.028	0.042
Kurtosis	0.60	−0.01
Skewness	0.89	0.87
Minimum	0.005	0.008
Maximum	0.078	0.123
CV	0.50	0.56
Count	94	83

****Statistically different between AN-BC vs AN-FC synapses. Mann–Whitney test *p* < 0.0001. Count refers to the total number of IMP-clusters analyzed

**Fig. 4:**
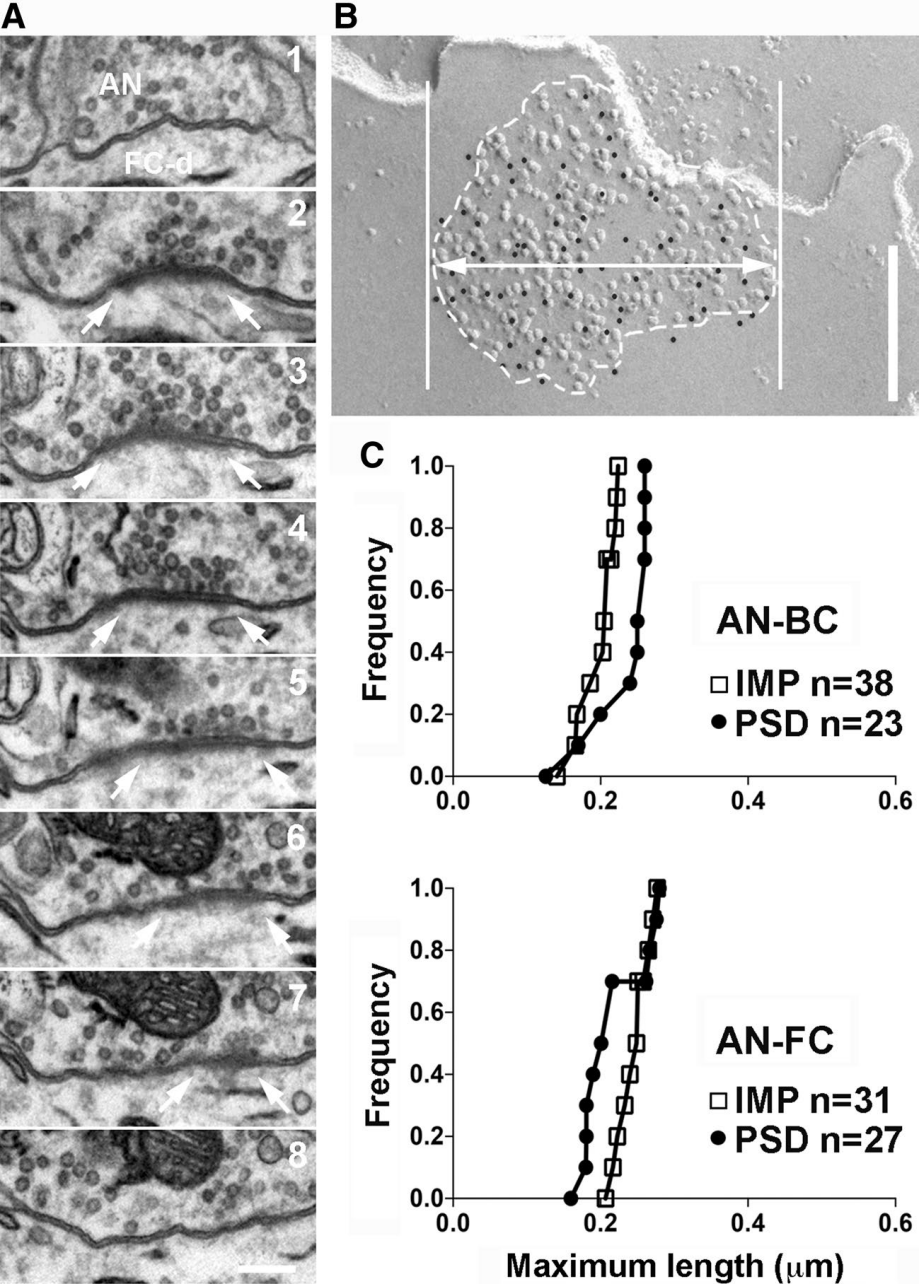
Size of the two types of AN synapses. **a** Serial ultrathin sections of an AN-FC synapse. *Arrows* indicate the edge of the PSD. *Scale bar* 200 nm. **b** FRIL image of the IMP-cluster of AN-FC synapse. The *thick line with a double arrow* indicates the maximum width of an IMP-cluster. *Scale bar* 200 nm. **c** Cumulative frequency plots of the maximum widths of the PSDs and IMP-clusters. The maximum widths of the PSD and IMP-clusters were not significantly different (AN-BC: *p* = 0.50, AN-FC: *p* = 0.5, Mann–Whitney *U* test)

We also analyzed the maximum widths of the PSDs from the AN-BC and AN-FC synapses in serial ultrathin sections to verify that the areas of the IMP clusters in the replicas was comparable to the areas of the PSDs visualized in the conventional ultrathin sections (Fig. 4). The maximum width of the PSDs from the AN-BC synapses on the cell body (median 0.26 μm, *n* = 23 synapses) and that of the AN-FC synapses on proximal dendrites (median 0.32 μm, *n* = 27 synapses) were not significantly different from the maximum width of the IMP clusters in each of the two types of synapses (Fig. 4); (maximum width of the IMP clusters for the AN-BC synapse, median 0.25 μm, *n* = 38 synapses, and *p* = 0.5; for AN-FC synapses, median 0.31 μm, *n* = 31 synapses, and *p* = 0.51; Mann–Whitney *U* test). Based on the results from two different analyses, the IMP clusters on the E-face correspond to PSDs, and the average synapse size of the two excitatory synapse types differs.

### Number and density of AMPAR subunits on AN-BC and AN-FC synapses

We used FRIL, which enables the reliable detection and precise localization of target proteins at a nanoscale spatial resolution with a very high labeling efficiency (Tanaka et al. 2005; Masugi-Tokita et al. 2007), to determine the distribution and number of GluA1–4, GluA3, and GluA4 subunits at AN-BC and AN-FC synapses. The distribution and quantity of GluA1–4, GluA3 and GluA4 subunits within the IMP cluster areas of mouse AN synapses varied between synapse types.

#### GluA1–4 (pan-AMPAR antibody labeling)

The distribution of the gold labeling and expression levels of GluA1–4 in the mouse AN-BC and AN-FC synapses were very similar to the previous findings in rats (Rubio et al. 2014). The GluA1–4 AMPAR immunogold particles appeared to be homogenously distributed throughout the IMP clusters of all AN-BC synapses and a majority of AN-FC synapses (Figs. 1, 2, 3). However, in some AN-FC synapses, AMPAR labeling was not uniformly distributed over the IMPs, and in some cases, IMP cluster sub-regions entirely lacked labeling (Fig. 2b–d**)**. Nevertheless, all IMP clusters of the AN-BC and AN-FC synapses displayed gold labeling for GluA1–4 (Table 3). As expected from the substantial variability in PSD areas, the number of gold particles per IMP cluster in both the AN-BC and AN-FC synapses was quite variable [coefficient of variation (CV) = 0.44 for AN-BC, CV = 0.59 for AN-FC]. In contrast, the average density of gold particles per IMP cluster was less variable (CV = 0.18 for AN-BC, CV = 0.39 for AN-FC).

**Table 3:** Total number of gold particles for GluA1–4 (pan-AMPAR), GluA3, and GluA4 at AN synapses, together with the total number of IMP-clusters

	AVCN	DCN
	AN-BC	AN-FC
GluA1–4		
Total gold	1232	1004
IMP cluster N (positive/total)	34/34	32/32
GluA3		
Total gold	734	231
IMP clusters N (positive/total)	30/30	23/23
GluA4		
Total gold	305	635
IMP clusters N (positive/N)	30/30	28/28

The mean numbers of immunogold particles for AMPARs in the AN-BC and AN-FC synapses were similar (*p* = 0.2; Mann–Whitney *U* test; Fig. 6a; Table 4). The area of the IMP clusters was significantly smaller in the AN-BC synapses than in the AN-FC synapses (Table 2); thus, the average density of AMPARs was significantly increased by more than 1.5-fold in the AN-BC synapses (*p* < 0.0001, Mann–Whitney *U* test; Fig. 6a; Table 4).

**Table 4:** Number and density of gold particles for GluA1–4, GluA3, and GluA4 at AN-BC and AN-FC synapses

	AN-BC synapses	AN-FC synapses
GluA1–4	34	32
IMP-clusters (*n*)		
Number of gold particles/IMP-cluster	36 ± 3	32 ± 3
Mean (±SEM)	35	26
Median	7–74	5–67
Range		
Density of gold particles/IMP-cluster (μm^2^)	1100 ± 35	623 ± 47
Mean (±SEM)	1034	623
Median	732–1571	223–1257
Range		
GluA3		
IMP-clusters (*n*)	30	23
Number of gold particles/IMP-cluster		
Mean (±SEM)	25 ± 2	10 ± 1
Median	21	9
Range	9–57	3–20
Density of gold particles/IMP-cluster (μm^2^)		
Mean (±SEM)	946 ± 50	184 ± 19
Median	954	179
Range	500–1556	36–381
GluA4		
IMP-clusters (*n*)	28	28
Number of gold particles/IMP-cluster		
Mean (±SEM)	10 ± 1.2	23 ± 3
Median	9.5	19
Range	1–22	5–44
Density of gold particles/IMP-cluster (μm^2^)		
Mean (±SEM)	337 ± 34	561 ± 38
Median	372	539
Range	47–727	108–1091

Both the AN-BC and AN-FC synapses exhibited a strong positive correlation between the number of labeled AMPARs and the IMP cluster area, consistent with the possibility that the AMPAR density is constant across synapses with the same connection type (Fig. 6b; AN-BC: *r* = 0.90; AN-FC: *r* = 0.47) and the number of AMPARs in individual synapses strongly depends on the size of the AN synapses.

#### GluA3

**Fig. 5:**
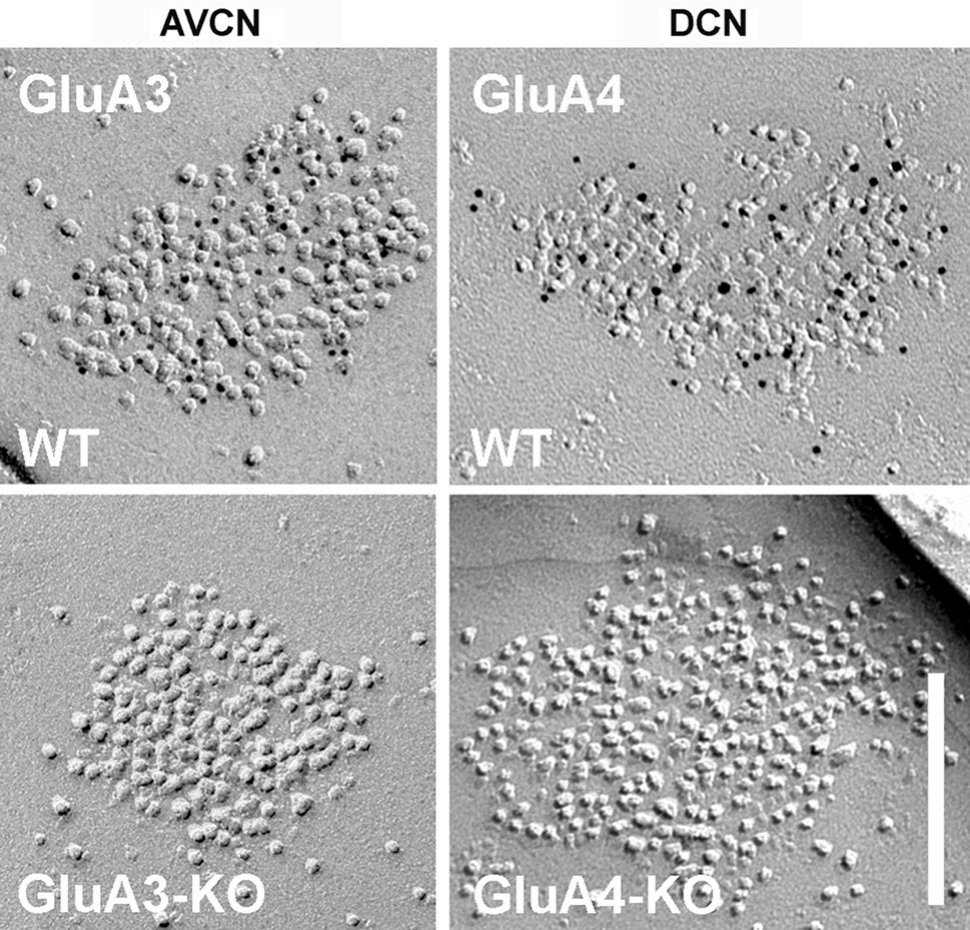
IMP-clusters of GluA3 and GluA4 knockout mice lack gold labeling for GluA3 and GluA4, respectively. GluA3 gold particles label IMP-clusters of AN-BC synapses of wild type (WT) but not GluA3 knockout mice in the anteroventral cochlear nucleus (AVCN). GluA4 gold particles label IMP-clusters of AN-FC synapses of wild type (WT) but not GluA4 knockout mice in the dorsal cochlear nucleus (DCN). *Scale bar* 200 nm

The GluA3 immunogold particles appeared to be fairly uniformly distributed over the IMP clusters of all AN-BC synapses, but not the AN-FC synapses, which exhibited less gold labeling (Fig. 3). Nevertheless, all IMP clusters of the AN-BC and AN-FC synapses were gold labeled for GluA3 (Table 3). As expected from the substantial range of PSD areas analyzed for both synapse types, the numbers of gold particles for GluA3 were highly variable (CV = 0.63 for AN-BC, CV = 0.47 for AN-FC); the average densities of gold particles per IMP cluster were less variable (CV = 0.5 for AN-BC, CV = 0.35 for AN-FC). The IMP clusters of the GluA3 KO mice lacked GluA3 immunogold particles, confirming the specificity of the antibody (Fig. 5).

**Fig. 6:**
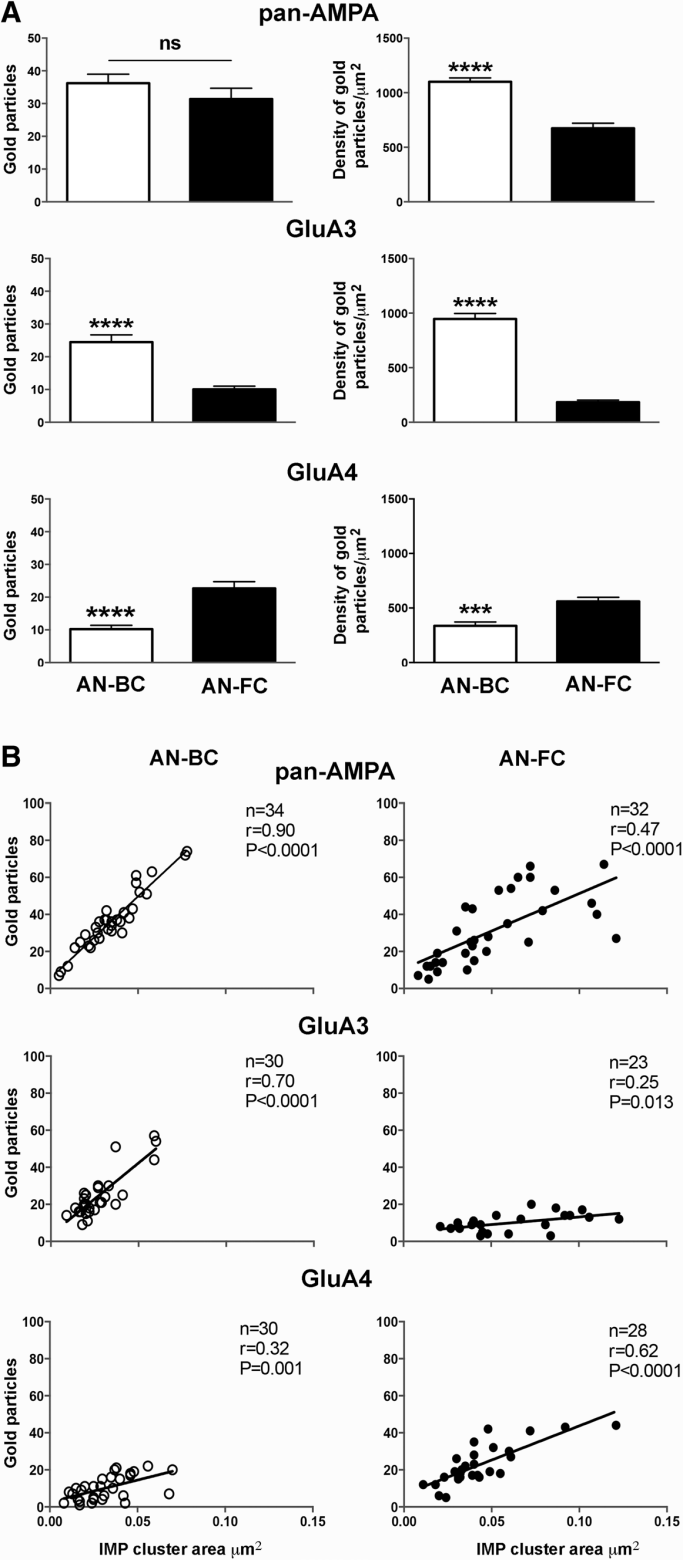
Number and density of AMPAR subunits in IMP-clusters of AN-BC and AN-FC synapses. **a** Histograms show the average density and number of gold particles per IMP-cluster for AMPAR, GluA3, and GluA4 at AN-BC and AN-FC synapses. The number of AMPAR gold particles was similar for AN-BC and AN-FC synapses, although the density was higher for AN-BC synapses. The number and density of GluA3 gold particles were higher for AN-BC synapses than for AN-FC synapses. The number and density of GluA4 gold particles were higher for AN-FC synapses than for AN-BC synapses [Mann–Whitney *U* test (*p* < 0.0001****; *p* = 0.0001***)]. **b** Correlation of the number of gold particles and IMP-cluster area. Scatterplots of the number of gold particles for GluA1–4; GluA3 and GluA4 vs. the IMP-cluster areas of AN-BC and AN-FC synapses (Spearman’s rank-order test)

The quantitative analysis of the gold labeling indicated a 2.5-fold increase in the level of the GluA3 subunit in the AN-BC synapses compared with that in the AN-FC synapses (*p* < 0.0001; Mann–Whitney *U* test; Fig. 6a; Table 4). The average density was increased more than fivefold in the AN-BC synapses (*p* < 0.0001, Mann–Whitney *U* test; Fig. 6a; Table 4).

The correlation between GluA3 labeling with the IMP cluster area was substantially stronger in the AN-BC synapses (*r* = 0.70) than the AN-FC synapses (*r* = 0.25), suggesting that the GluA3 density was only constant across AN-BC synapses.

#### GluA4

The GluA4 immunogold particles appeared to be evenly distributed over the IMP clusters of all AN-BC and AN-FC synapses; however, less gold labeling was observed on AN-BC synapses (Fig. 3). Nevertheless, all IMP clusters of both synapse types were labeled for GluA4 (Table 3). As expected from the substantial variability in the PSD areas, the numbers of labeled GluA4 subunits were also highly variable among the AN-BC and AN-FC synapses (CV = 0.49 for AN-BC, CV = 0.48 for AN-FC); the average densities of the gold particles per IMP cluster were less variable (CV = 0.28 for AN-BC, CV = 0.27 for AN-FC). The IMP clusters of the GluA4 KO mice lacked GluA4 immunogold particles, confirming the specificity of the antibody (Fig. 5).

According to the quantitative analysis of the gold labeling, AN-FC synapses contain ~2.3-fold more GluA4 than AN-BC synapses (*p* < 0.0001; Mann–Whitney *U* test; Fig. 6a; Table 4). The average density of GluA4 subunits was also ~1.7-fold higher at the AN-FC synapses than the AN-BC synapses (*p* < 0.0001, Mann–Whitney *U* test; Fig. 6a; Table 4).

The correlation between GluA4 labeling with the IMP cluster area was substantially stronger in the AN-FC synapses (*r* = 0.62) than the AN-BC synapses (*r* = 0.32) (Fig. 6b), suggesting that the GluA4 density was only constant across AN-FC synapses.

### Intrasynaptic distribution of AMPAR subunits on AN-BC and AN-FC synapses

We subsequently determined the two-dimensional distribution of AMPAR subunits along the postsynaptic plasma membrane using FRIL. The intrasynaptic distribution of AMPARs (GluA1–4), GluA3, and GluA4 relative to the border of demarcation was performed by defining six divisions (D1–D5, from the periphery to center, and outer rim division) as previously described (Budisantoso et al. 2012, 2013).

#### GluA1–4 (pan-AMPAR antibody labeling)

**Fig. 7:**
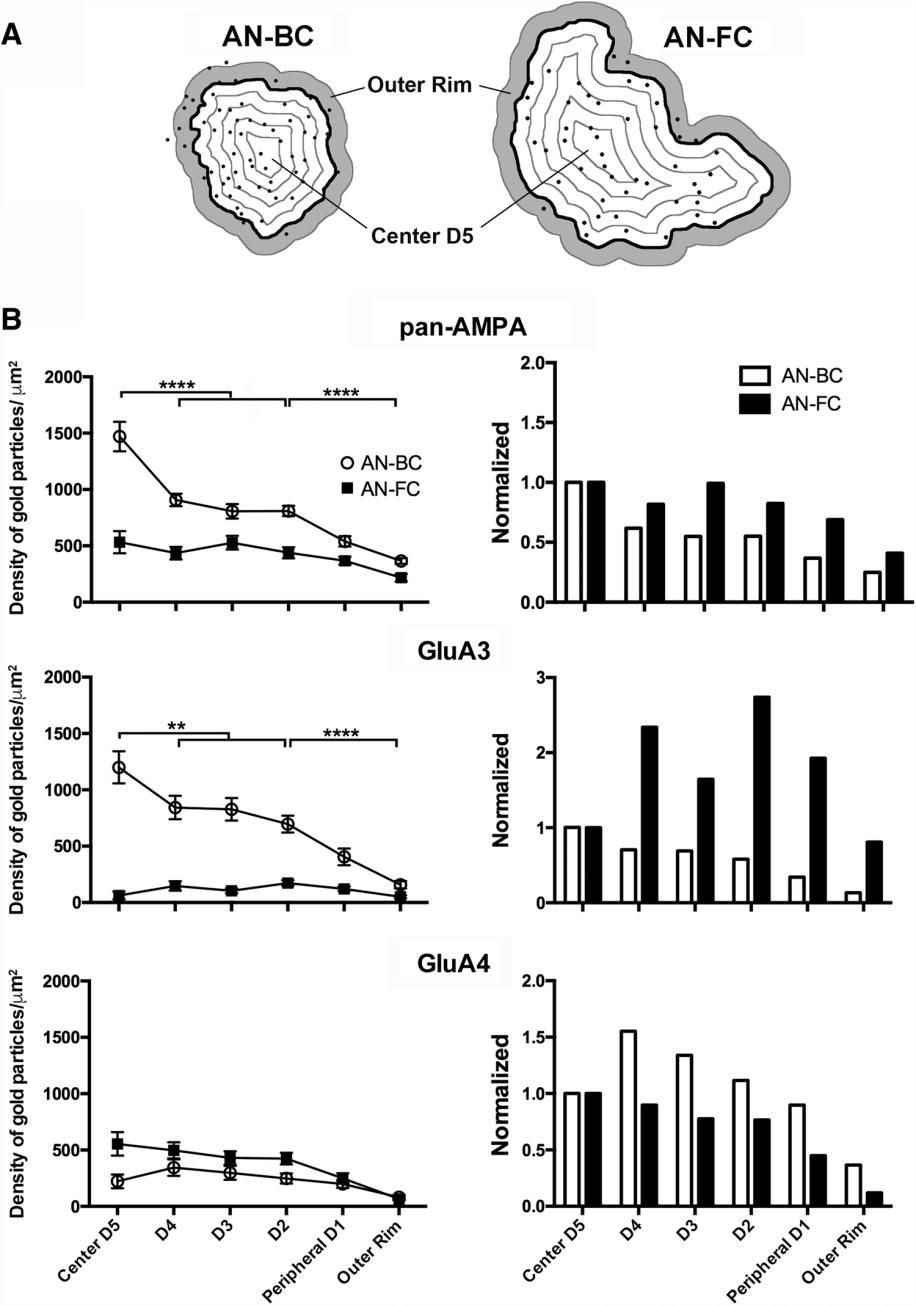
Intrasynaptic distribution of AMPAR subunits within IMP-cluster area of auditory nerve (AN) synapses on bushy (BC) and fusiform (FC) cells. **a** Drawings of two heat maps corresponding to AN synapses on BCs and FCs to show the five divisions demarcation. *Thick line* represents the outline of the PSD. *Black dots* correspond to the immunogold particles. **b** Histograms on the *left* show the intrasynaptic distribution for pan-AMPAR, GluA3, and GluA4 gold labeling in six concentric divisions from the center (*D5*) to the peripheral edge (*D1; black thick line* on the schematic drawing) of the IMP-cluster; the labeling on the outer rim (*gray* on the schematic drawing) of the IMP-cluster is also shown. Only statistically different comparisons are indicated. Number of IMP-cluster analyzed, pan-AMPAR: AN-BC *n* = 21, AN-FC *n* = 20; GluA3: AN-BC *n* = 22, AN-FC *n* = 20; GluA4: AN-BC *n* = 20, AN-FC *n* = 16. Statistical comparisons are shown only for AN-BC synapses, *p* < 0.0001****, *p* < 0.01**, one-way ANOVA. Histograms on the right show normalized data

**Fig. 8:**
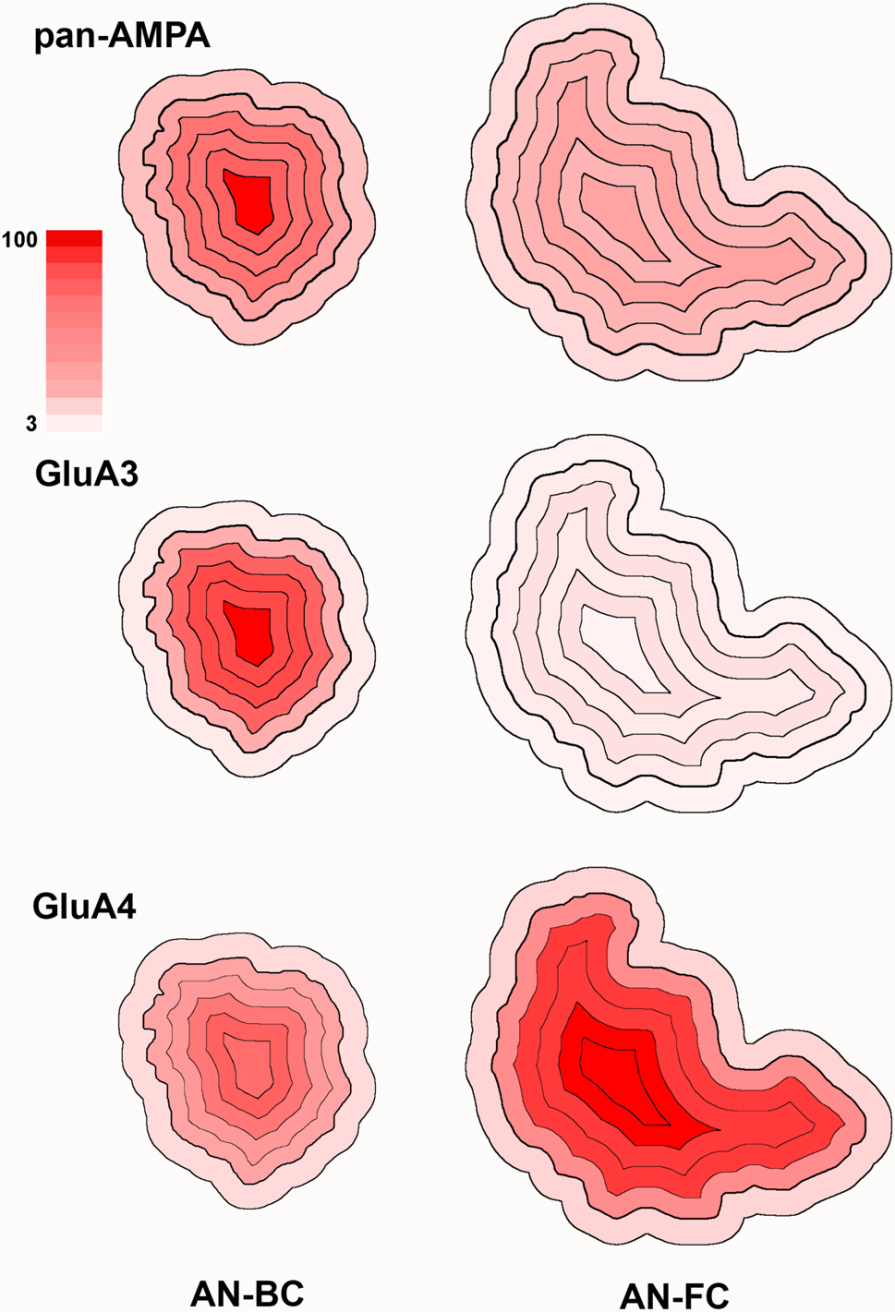
Heat maps of the intrasynatic distribution of pan-AMPAR, GluA3, and GluA4 gold labeling at IMP-clusters of AN synapses on bushy and fusiform cells of wild-type mice. The heat maps represent the labeling density in individual subdivisions relative to the highest labeling density per antibody across AN-BC and AN-FC synapses (Fig. 7). *Color scale* 100% *red*, 3% *pale pink*

At the AN-BC synapses, gold particles were preferentially distributed towards the center of the IMP cluster and decreased towards the peripheral edge of the PSD (Figs. 7, 8). The highest density of gold particles was observed in the central division (D5, 1470 particles/μm^2^), followed by the adjacent D4 division (907 particles/μm^2^) and the other divisions (*p* < 0.0001, one-way ANOVA). The most peripheral division of the IMP cluster (D1, 540 particles/μm^2^) and the outer rim division (365 particles/μm^2^) exhibited the lowest density (*p* < 0.0001, one-way ANOVA). Of the 21 IMP clusters analyzed, 17 clusters (81%) exhibited a peak distribution at the center.

At the AN-FC synapses, the distribution of gold particles for GluA1–4 within the IMP cluster was relatively homogenous (Figs. 7, 8). The differences in the densities between divisions within the IMP cluster were insignificant, with the exception of the outer rim division, which exhibited a significantly lower density (217 particles/μm^2^, *p* < 0.01, one-way ANOVA).

#### GluA3

At the AN-BC synapses and as observed with the pan-AMPAR immunolabeling, the central division exhibited the highest concentration of gold particles (Figs. 7, 8). Significantly less gold labeling was observed towards the peripheral divisions of the PSD. The difference between the central density (D5, 1250 particles/μm^2^) and the adjacent D4 division (840 particles/μm^2^) was significant (*p* < 0.001, one-way ANOVA). The most peripheral division (D1) exhibited the lowest density (405 particles/μm^2^; *p* < 0.001, one-way ANOVA). Of the 22 IMP clusters analyzed, 15 clusters (68%) exhibited this peak central distribution of GluA3 immunogold particles.

At the AN-FC synapses, a low density of immunogold particles was observed throughout the subdivisions (Figs. 7, 8). Based on the intrasynaptic distribution, the gold particle density was apparently increased at locations away from the very center of the synapse.

#### GluA4

At the AN-BC synapses, the GluA4 labeling was relatively low in all divisions of the IMP cluster (Figs. 7, 8). According to the analysis of the intrasynaptic distribution, the density of gold particles was similar from the center to the outer rim of the IMP cluster (*p* > 0.5, one-way ANOVA), indicating a relatively homogenous distribution.

At the AN-FC synapses, the intrasynaptic distribution of gold particles was relatively homogeneous within the central divisions (D5-D2) of the PSD (Figs. 7, 8). The most peripheral division (249 particles/μm^2^) and the outer rim division (66 particles/μm^2^) of the IMP cluster exhibited a significantly lower density than the other divisions (*p* < 0.0001, one-way ANOVA).

### Intrasynaptic distribution of AMPAR subunits on AN-BC synapses from WT and GluA3 KO mice

**Fig. 9:**
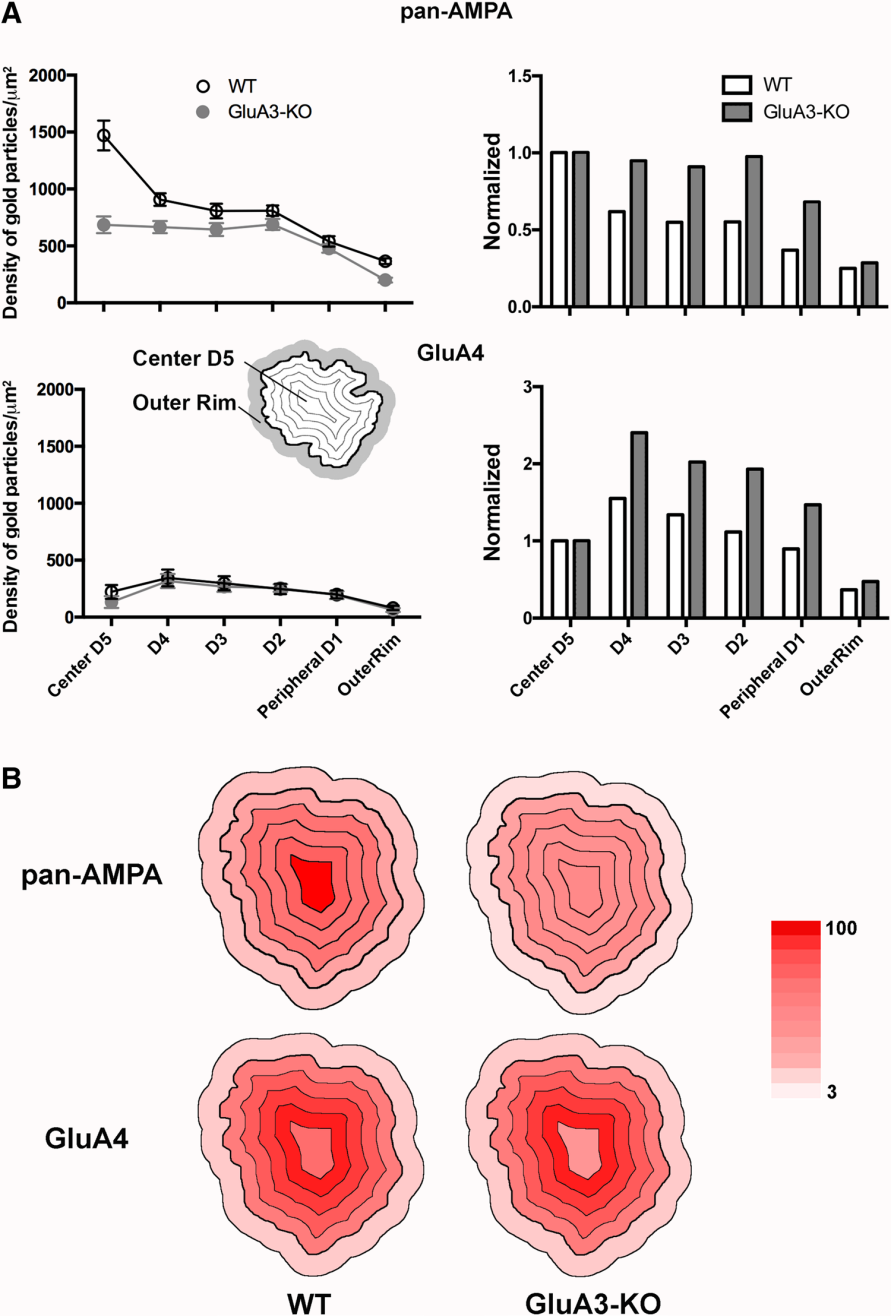
Intrasynaptic distribution of AMPAR subunits within IMP-cluster area of auditory nerve synapses on bushy cells of WT and GluA3-knockout mice. **a** Histograms on the *left* show the intrasynaptic distribution for pan-AMPAR and GluA4 gold labeling on six concentric areas from the center (*D5*) to the peripheral edge (*D1; black thick line* on the schematic drawing) of the IMP-cluster; the labeling on the outer rim (*gray* on the schematic drawing) of the IMP-cluster is also shown. Number of IMP-cluster analyzed, pan-AMPAR: wild type (WT) *n* = 21, GluA3-KO *n* = 35; GluA4: WT *n* = 20, GluA3-KO *n* = 15. AN-BC synapses of WT and GluA3-KO labeled with pan-AMPA: ***p < 0.001; F:36.34; simple two-way ANOVA. Histograms on the *right* show the data normalized by the value in *D5* for each genotype. **b** Heat maps of the intrasynaptic distribution of pan-AMPAR and GluA4 gold labeling of AN synapses on bushy cells of wild-type (WT) and GluA3-KO mice. The heat maps represent labeling densities at individual subdivisions relative to the highest labeling density for each antibody across WT and GluA3-KO mice. *Color scale* 100% (maximum labeling) *red*, 3% *pale pink*

The pan-AMPAR and GluA3 immunogold particles located within the IMP cluster were concentrated at the center of AN-BC synapses. We analyzed the intrasynaptic distribution of GluA1–4 and GluA4 immunogold labeling in GluA3 KO mice to determine whether GluA3 contributed to this central distribution (Fig. 9).

#### GluA1–4 immunolabeling

The GluA1–4 labeling at the AN-BC synapses was relatively homogeneous in the GluA3 KO mice (Fig. 9). The differences in the densities between the 5 divisions of the IMP cluster were insignificant (p > 0.5, one-way ANOVA). The peripheral D1 and the outer rim divisions had the lowest density of gold labeling in the WT mice (D1: 477.4 particles/μm^2^, outer rim: 200 particles/μm^2^; *p* < 0.01, one-way ANOVA). The intrasynaptic distribution of GluA1–4 on the AN-BC synapses from the WT and GluA3 KO mice exhibited a significant difference (*p* < 0.001; F: 36.34; simple two-way ANOVA). Based on these findings, AMPARs within the IMP cluster are not concentrated at the center of AN-BC synapses.

#### GluA4

The distribution of the GluA4 immunogold particles at the AN-BC synapses was similar in the GluA3 KO and WT mice (Fig. 9), suggesting that the lack of GluA3 does not affect the distribution of the GluA4 subunits within the PSD. Moreover, GluA4 does not compensate for the lack of GluA3.

## Discussion

### Differences in AMPARs at AN synapses

Not all presynaptic action potentials produce postsynaptic action potentials, and the filtering and selection of signals occur at synapses. The amplitudes and kinetics of postsynaptic responses are profoundly controlled by the subunit composition of postsynaptic receptors. The subunit composition may be differentially regulated at individual synapses to enable optimized information processing. Auditory information processing depends on the high frequency and precise timing of action potential firing of auditory relay neurons. Thus, GluA2-lacking AMPARs with high Ca^2+^ permeability and submillisecond gating kinetics are prominently expressed in many auditory relay neurons (Gardner et al. 2001).

Of the four AMPAR subunits, GluA3 and GluA4 have rapid gating kinetics. Using postembedding immunogold labeling, AN synapses on BCs and FCs of the cochlear nucleus were shown to contain GluA2/3 and GluA4 subunits (Rubio and Wenthold 1997; Wang et al. 1998; Whiting et al. 2009). However, these studies only analyzed the presence of AMPAR subunits at the synapse. Furthermore, the level of GluA3 expression was not determined, because the applied antibody recognized both GluA2 and GluA3 subunits. A quantitative analysis of the expression patterns of GluA3 subunits at the AN-BC and AN-FC synapses has not been performed. Specific antagonists for AMPAR subunits are not available; thus, the roles of GluA3 and GluA4 in mediating synaptic transmission at AN synapses are unknown. However, differences in the kinetics of the synaptic responses at the two synapses have been identified (Gardner et al. 1999, 2001). Synaptic transmission is extremely fast and reliable at AN-BC synapses, thus preserving the information contained in the timing of AN spikes (Gardner et al. 1999; Fujino and Oertel 2003). Synaptic transmission is significantly slower at AN-FC synapses than at AN-BC synapses (Gardner et al. 1999, 2001). This difference in kinetics may arise from a combination of the differences in AMPAR density, the central organization of AMPARs, and a GluA3- or GluA4-dominant subunit composition (Geiger et al. 1995). Our study provides novel insights into the differences in kinetics between the GluA3 and GluA4 subunits.

Using FRIL and a pan-AMPAR (GluA1–4) antibody with superb labeling efficiency for functional AMPAR channels in FRIL samples (Tanaka et al. 2005), mouse AN synapses on BCs and FCs contain similar numbers of gold particles for GluA1–4, approximately 36 and 32, respectively. A similar number of gold particles for GluA1–4 was also identified in the rat (Rubio et al. 2014). Using specific antibodies against GluA3 and GluA4, AN-BC and AN-FC synapses were shown to contain both subunits. Interestingly, AN-BC synapses express more GluA3 subunits, whereas AN-FC synapses express more GluA4 subunits. The labeling efficiency of the GluA3 and GluA4 antibodies is unknown; however, on average, 25 gold particles for GluA3 were observed at AN-BC synapses, whereas only 10 gold particles were observed at AN-FC synapses. Ten GluA4 gold particles were observed at AN-BC synapses compared with 22 particles at AN-FC synapses. Thus, GluA3 is the main fast gating AMPAR subunit present in the ultrafast AN-BC synapse, whereas GluA4 is the main subunit present in the AN-FC synapse, which is a slower synapse.

### Intrasynaptic distribution of GluA3 and GluA4 subunits

The subsynaptic localization of AMPARs is an important aspect of PSD organization (MacGillavry et al. 2011, 2013). According to computational studies, the nanoscale organization of AMPARs and the location of glutamate release may impact the quantal synaptic response (Franks et al. 2003; Raghavachari and Lisman 2004). Postsynaptic AMPARs that directly oppose the presynaptic release site are likely to be exposed to high concentrations of glutamate. Therefore, the local density of receptors close to the release site would affect the strength of the postsynaptic response (Franks et al. 2003; MacGillavry et al. 2013; Lisman et al. 2007). However, the situation may not be this simple, because simulation studies also show that the AMPAR response and glutamate concentration are not linearly correlated and AMPARs that immediately face the release site may become saturated (Tarusawa et al. 2009). Moreover, glutamate release that occurs slightly offset from AMPAR clusters would also produce a sizable response because of the additive responses from intermediately activated AMPARs (Tarusawa et al. 2009). The number of these intermediately activated AMPARs is also expected to decrease as the release site is shifted to the periphery of the synaptic specialization. The most efficient synaptic signaling would be achieved when presynaptic release and the postsynaptic AMPARs are concentrated at the center of synapses.

Live-cell super-resolution imaging studies of the PSD of cultured hippocampal neurons have indicated that receptors are not uniformly distributed within the PSD and are typically confined within the subsynaptic domains (Kerr and Blanpied 2012; MacGillavry et al. 2013; Tang et al. 2016). One study examined the organization of AMPAR subunits (GluA2/3, GluA4, and GluA2) at AN-BC synapses (Wang et al. 1998) and observed a homogenous distribution of receptors within the PSD. However, only one ultrathin section was analyzed per PSD. FRIL cannot capture the dynamics of AMPAR movement; however, the distribution of the molecules revealed by FRIL using fixed brain slices may be considered a two-dimensional snapshot of the AMPAR distribution at a specific time point. FRIL has been used to investigate the intrasynaptic distribution of AMPARs at the calyx of Held synapse on neurons from the medial nucleus of the trapezoid body (MNTB) (Budisantoso et al. 2013) and reticulogeniculate synapses in the dLGN (Budisantoso et al. 2012). In these synapses, AMPARs are homogenously distributed throughout the PSD, and limited gold labeling is only observed at the most peripheral edge. Based on our data, the distribution of AMPAR gold labeling at AN-FC synapses is relatively similar to that at calyceal and reticulogeniculate synapses, whereas the distribution at AN-BC synapses differs, because the immunogold particles concentrate at the center. A tendency toward central organization was also identified in the retinogeniculate synapse; thus, central organization and a homogeneous distribution may not be two completely separate states, and a continuum may exist in the organization of these receptors. The central organization was identified in 81% of the AN-BC synapses analyzed, suggesting that this peak distribution pattern is not random.

The intrasynaptic distribution of AMPARs may depend on the subunit composition. Here, AN synapses on BCs contained significantly higher levels of GluA3 (2.5-fold more gold particles) than AN-FC synapses. The GluA3 subunit may exhibit the central organization pattern. The signature central organization of the AMPARs distributed at AN-BC synapses is lost in the GluA3 KO mice. Regarding other CNS synapses, GluA3 expression levels at reticulogeniculate synapses and the calyx of Held synapse have not yet been determined. However, according to an electrophysiology study, GluA4 is the main AMPAR subunit expressed at the calyx of Held synapse (Yang et al. 2011). The authors showed that the amplitude and frequency of the excitatory postsynaptic currents (EPSCs) are decreased in GluA4 KO mice, but not in GluA3 KO mice. GluA4-dominant synapses may exhibit a non-central and homogenous organization of AMPARs. A recent study in the hippocampus using postembedding immunogold labeling with a different antibody from the antibody used in the study of GluA3 in serial ultrathin sections also observed a more central localization of GluA3 the PSD than GluA1 (Jacob and Weinberg 2015). GluA1 was located closer to the edge of the synapse. Thus, high levels of GluA3 at a specific synapse may determine the organization of AMPARs within the center of the PSD.

### Functional implications of the intrasynaptic distribution

AN-BC synapses are approximately half the size of AN-FC synapses and have a higher density of fast gating GluA3 AMPAR subunits, which appear to be concentrated at the center of the synapse. The kinetics of the AMPAR channels may be faster at smaller synapses, as fewer AMPARs would be exposed to low concentrations of glutamate. Based on simulations, the average rise time of AMPAR-mediated postsynaptic responses correlated with the synaptic area (Tarusawa et al. 2009); however, the effect was relatively small, and the postsynaptic response decay time was predominately determined by the deactivation kinetics of AMPARs, which are independent of the glutamate concentration. Similar to AN synapses, retinogeniculate synapses are also approximately half the size of corticogeniculate synapses. However, the synapses contain similar numbers of AMPARs, implying that retinogeniculate synapses have a higher density of AMPARs. The response amplitude in synapses with a higher AMPAR density is expected to be slightly higher (Tarusawa et al. 2009). The variability of the response that arises from many other sources, such as synapses of the same size, may obscure this modest difference in the quantal response amplitude caused by the difference in the AMPAR density. Consistent with this hypothesis, the quantal responses from retinogeniculate and corticogeniculate synapses observed in electrophysiological recordings were not different (Tarusawa et al. 2009).

An important but unresolved question is whether the presynaptic release site is aligned with postsynaptic receptor clusters. In addition to the role of the intrasynaptic receptor distribution in the synaptic response per se, peripheral receptors may tend to exhibit lateral diffusion. Therefore, synapses that require high-fidelity transmission with no or low plasticity may concentrate receptors in the center of the synapse to avoid the loss of receptors due to lateral diffusion. Further characterization of the specific anatomical and molecular organization of presynaptic release sites and postsynaptic AMPARs within AN synapses on BCs and FCs is required to understand how the synapses are tuned for optimal central sound processing.

## Electronic supplementary material

Below is the link to the electronic supplementary material.

Supplemental Figure 1. Immunoblots for anti GluA3 and GluA4 antibodies. Characterization of GluA3 and GluA4 knockout mice. Immunoblots of GluA3 and GluA4. **A-D**. Production of Gria3- and Gria4-flox mice. (**A-B)** Schematic representations of Gria3- and Gria4 genomic DNA (*Gria3* ^+^ and *Gria4* ^+^) and targeted genomes (*Gria3* ^flox^ and *Gria4* ^flox^). The closed boxes indicate the coding exons. The filled ovals in the targeted genomes delineate the 5’ and 3’ termini of the targeting vectors. The blue and red bars indicate probe regions (5’, neo and 3’) for Southern blot analysis. Two *frt* sequences (semicircles) are attached to remove the neomycin resistant gene (Neo). Triangles indicate *loxP* sequences. pA, polyadenylation signal sequence; IRES, internal ribosome entry site; SD, splice donor sequence. **(C-D)** Southern blot analysis of genomic DNA prepared from wild-type ES cells (C57BL/6) and targeted clones (No. 183-J13 for *Gria3* ^flox/+^ and No. 206-B7 for *Gria4* ^flox/+^). Positions of DNA size markers (kb) are indicated to the left. **E**. The 100 kDa band corresponding to GluA3 is indicated with an arrow head (lanes 1 and 3, respectively). Asterisks (*) indicate non-specific bands cross-reacting with the anti-GluA3 antibody, because they are detected in cytosolic fraction (lanes 2 and 4, respectively). **B**. 100 kDa band marked by an arrowhead corresponds to GluA4. The bands marked by asterisks are detected in cytosolic fraction, demonstrating that they are a consequence of a non-specific interaction (TIF 797 KB)
