# Supplementary figures and images for: The number and distribution of AMPA receptor channels containing fast kinetic GluA3 and GluA4 subunits at auditory nerve synapses depend on the target cells
Source: Brain Struct Funct. 2017 Apr 10;222(8):3375–93. doi: 10.1007/s00429-017-1408-0 (PMC5676837; doi:10.1007/s00429-017-1408-0)

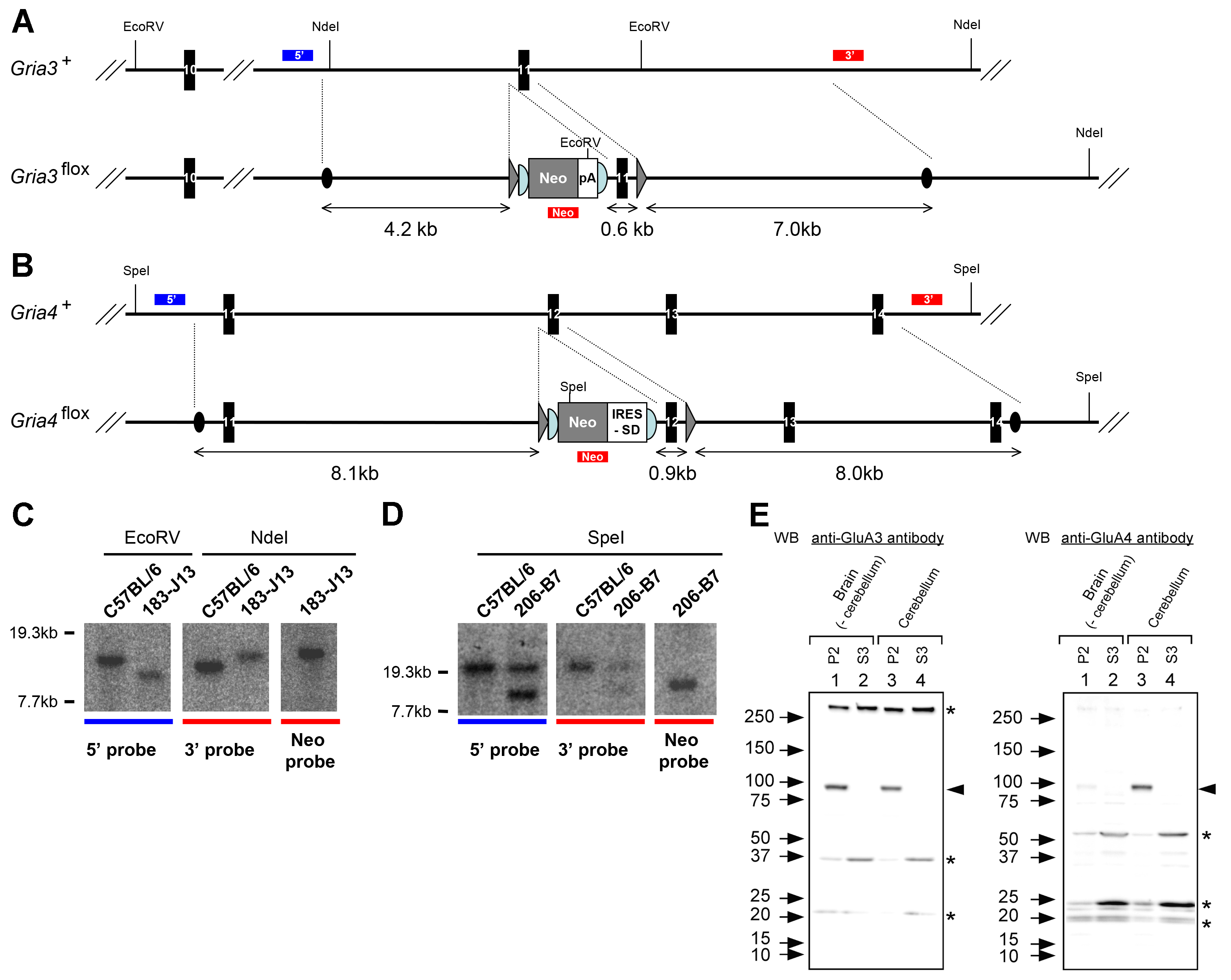

Supplement: Supplementary file 1 — Supplemental Figure 1. Immunoblots for anti GluA3 and GluA4 antibodies. Characterization of GluA3 and GluA4 knockout mice. Immunoblots of GluA3 and GluA4. A-D. Production of Gria3- and Gria4-flox mice. (A-B) Schematic representations of Gria3- and Gria4 genomic DNA (Gria3 + and Gria4 +) and targeted genomes (Gria3 flox and Gria4 flox). The closed boxes indicate the coding exons. The filled ovals in the targeted genomes delineate the 5’ and 3’ termini of the targeting vectors. The blue and red bars indicate probe regions (5’, neo and 3’) for Southern blot analysis. Two frt sequences (semicircles) are attached to remove the neomycin resistant gene (Neo). Triangles indicate loxP sequences. pA, polyadenylation signal sequence; IRES, internal ribosome entry site; SD, splice donor sequence. (C-D) Southern blot analysis of genomic DNA prepared from wild-type ES cells (C57BL/6) and targeted clones (No. 183-J13 for Gria3 flox/+ and No. 206-B7 for Gria4 flox/+). Positions of DNA size markers (kb) are indicated to the left. E. The 100 kDa band corresponding to GluA3 is indicated with an arrow head (lanes 1 and 3, respectively). Asterisks (*) indicate non-specific bands cross-reacting with the anti-GluA3 antibody, because they are detected in cytosolic fraction (lanes 2 and 4, respectively). B. 100 kDa band marked by an arrowhead corresponds to GluA4. The bands marked by asterisks are detected in cytosolic fraction, demonstrating that they are a consequence of a non-specific interaction (TIF 797 KB) [file 429_2017_1408_MOESM1_ESM.tif]
